# Big catch, little sharks: Insight into Peruvian small-scale longline fisheries

**DOI:** 10.1002/ece3.1104

**Published:** 2014-05-14

**Authors:** Philip D Doherty, Joanna Alfaro-Shigueto, David J Hodgson, Jeffrey C Mangel, Matthew J Witt, Brendan J Godley

**Affiliations:** 1 Centre for Ecology and Conservation School of Biosciences, University of Exeter, Penryn CampusPenryn, Cornwall, TR10 9EZ, U.K; 2 Environment and Sustainability Institute, University of Exeter, Penryn CampusPenryn, Cornwall, TR10 9EZ, U.K; 3 Pro DelphinusOctavio Bernal 572-5, Lima 11, Peru

**Keywords:** Conservation, CPUE, Peru, sharks, small-scale fisheries, sustainability

## Abstract

Shark take, driven by vast demand for meat and fins, is increasing. We set out to gain insights into the impact of small-scale longline fisheries in Peru. Onboard observers were used to document catch from 145 longline fishing trips (1668 fishing days) originating from Ilo, southern Peru. Fishing effort is divided into two seasons: targeting dolphinfish (*Coryphaena hippurus*; December to February) and sharks (March to November). A total of 16,610 sharks were observed caught, with 11,166 identified to species level. Of these, 70.6% were blue sharks (*Prionace glauca)*, 28.4% short-fin mako sharks (*Isurus oxyrinchus*), and 1% were other species (including thresher (*Alopias vulpinus*), hammerhead (*Sphyrna zygaena*), porbeagle (*Lamnus nasus*), and other Carcharhinidae species (*Carcharhinus brachyurus*, *Carcharhinus falciformis*, *Galeorhinus galeus*). Mean ± SD catch per unit effort of 33.6 ± 10.9 sharks per 1000 hooks was calculated for the shark season and 1.9 ± 3.1 sharks per 1000 hooks were caught in the dolphinfish season. An average of 83.7% of sharks caught (74.7% blue sharks; 93.3% mako sharks) were deemed sexually immature and under the legal minimum landing size, which for species exhibiting k-selected life history traits can result in susceptibility to over exploitation. As these growing fisheries operate along the entire Peruvian coast and may catch millions of sharks per annum, we conclude that their continued expansion, along with ineffective legislative approaches resulting in removal of immature individuals, has the potential to threaten the sustainability of the fishery, its target species, and ecosystem. There is a need for additional monitoring and research to inform novel management strategies for sharks while maintaining fisher livelihoods.

## Introduction

There is growing concern regarding the rate of decline of the world's shark populations due to overfishing (Stevens et al. 2000; Baum et al. 2003; Worm et al. 2013). Additionally, sharks caught as bycatch represent approximately 50% of all chondrichthyan fish catch globally (Bonfil 1994; Stevens et al. 2005). It has been suggested that more than half of all chondrichthyans and three-quarters of pelagic shark species are predicted to be threatened or near threatened (Clarke et al. 2006; Dulvy et al. 2008, 2014), highlighting the need for management programs to enhance sustainability (Stevens et al. 2000).

Sharks are generally considered apex predators of the ecosystems in which they inhabit (Kitchell et al. 2002). Removal of sharks can result in trophic cascades, causing a shift to smaller mesopredators, which in turn can have a large impact on lower trophic levels (Kitchell et al. 2002; Myers et al. 2007; Heithaus et al. 2008). Sharks exhibit K-selected life history strategies, which are characterized by slow growth, late sexual maturity, low fecundity, long gestation periods, and extended life spans (Hutchings et al. 2012). These traits can make sharks more susceptible to exploitation than faster growing, more fecund fish species (Kitchell et al. 2002; Myers et al. 2007). Maximum per capita population growth rate (*r*_max_) and thus recovery potential of chondrichthyans have been shown to be significantly lower (reflecting increased extinction risk) than those of teleosts (Hutchings et al. 2012).

Most studies of shark fishing have, to date, focused on global catch at an industrial level and on associated bycatch of sharks in other fisheries, resulting in a paucity of information regarding direct take in small-scale and artisanal fishing operations. Fisheries and aquaculture directly employ over 44 million people worldwide, 98% of whom live in developing countries (Béné et al. 2012). Landings by small-scale fisheries (SSF) are thought to contribute up to a third of global catch (Chuenpagdee et al. 2006) and constitute a vital source of protein for approximately two billion people (Béné et al. 2012), especially within developing nations. Studies of SSF are, however, generally less numerous than those researching industrialized fishing activities (Chuenpagdee et al. 2006; Alfaro-Shigueto et al. 2010) and by their nature (i.e., remote, dispersed, and with limited enforcement) are very difficult to monitor, characterize, and manage (Chuenpagdee et al. 2006). Chondrichthyans constitute an important fishery resource for developing countries, with catches increasing by approximately 600% between 1950 and 2000 (Catarci 2004).

The southeastern Pacific Ocean off the coast of Peru, incorporating the Humboldt Current System, is one of the most productive coastal upwelling systems in the world (Carr 2002). Year-round upwelling attracts many species and supports the world's largest anchovy (*Engraulis ringens)* fishery (Bouchon et al. 2000). There are also extensive SSFs within this region, upon which more than 500,000 people are dependent, four times greater than the number dependent upon industrial fishing (Comision Permanente del Pacifico Sur CPPS 2003). Peru is one of the world's leading fishing nations (Vanuccini 1999); however, the reported catch within the elasmobranch fishery has been shown to represent a minor component of total landings (Stevens et al. 2000). Anchovies make up the majority of tonnage landed; this is used primarily in fishmeal, while sharks are a more important component with regard to human consumption (Alfaro-Shigueto et al. 2010).

Shark landings in Peru are regulated by the Ministry of Fisheries through the establishment of minimum landing sizes (MLS) for some elasmobranch species (Diario Oficial El Peruano 2001; Decreto Supremo N° 012-2001-PE; blue sharks (*Prionace glauca)*: 160 cm total length; short-fin mako sharks (*Isurus oxyrinchus;* herein mako): 170 cm total length). Enforcement of these regulations, however, has not been fully implemented, and awareness of these regulations among fishermen is still limited (Gilman et al. 2008). In an attempt to reduce the catch of dolphins within gillnet fisheries (Reyes 1993), and partly due to the collapse of traditional fisheries for bony fish (Bonfil 1994; Catarci 2004), longline fishing for sharks was reintroduced in Peru in the late 1980s and has greatly increased in recent years (Alfaro-Shigueto et al. 2010). Peru has no specific shark finning regulations and has no apparent current need for such regulations because both shark meat and fins are landed and commercialized, with demand coming from both domestic (Gilman et al. 2008; Alfaro-Shigueto et al. 2010) and international markets (PROMPEX Peru 2006). It is thought, however, that the domestic market for fresh shark meat underpins the industry in Peru more than the fin price (Gilman et al. 2008). The purpose of the current study was to characterize the Peruvian longline fishery and to evaluate the composition of shark catch through the use of onboard observers, in order to look toward promoting long-term fishery sustainability.

## Methods

From 2005 to 2010, we collected data from Ilo, a port involved in longline fishing, situated in the south of Peru (17°38′S, 71°20′W). Vessels in this fishery are defined as “small-scale” which, according to Peruvian fisheries regulations, contains boats with a maximum of 32.6 m^3^ of storage capacity, less than 15 m in length, and principally based on manual fishing techniques throughout fishing operations (El Peruano, Ley General de Pesca, 2001b). There are two distinct seasons, one targeting sharks (March to November) and another targeting dolphinfish (*Coryphaena hippurus*; December to February). While vessels fish year-round, different techniques and gear characteristics (leader material, hook size, branchline material, and length) are employed during the different seasons and are two distinct fisheries and are therefore considered separately (Alfaro-Shigueto et al. 2010).

Onboard observers were used to monitor fishing activity and were trained in shark species identification and in the collection of biometric measurements. In order to maximize data collection opportunities, onboard observers did not participate in fishing activity. The observers recorded fishing effort (number of sets, number of hooks, and length of trip) and the GPS location of fishing sets, taken at the start of the set and at the commencement of hauling in the hooks. Fork length was measured using a flexible measuring tape along with identification of species and sex. Shore-based observers were also used to gather information on number of trips departing from the port, length of trips, target species, and fishing grounds used. Observers worked throughout the year in order to sample from both fishing seasons and to monitor any changes in fishing effort, catch or spatial patterns within seasons (for additional description of methods, see Alfaro-Shigueto et al. 2010). A total of 84 observed trips comprising 618 sets of 462,438 hooks targeted sharks (58%), with 61 observed trips comprising 402 sets of 283,446 hooks targeting dolphinfish (42%; Table S2), totaling 1668 fishing days.

During both seasons, a range of large, J hooks were used (J1 (TL = 91 mm, gape = 30 mm) to J5 (TL = 57.7 mm, gape = 19.6 mm); Table S7), with larger (J1–J2) and fewer hooks spaced further apart when targeting sharks. Branchlines used were typically made of nylon multifilament cord. Cable leaders were used during shark season due to their improved ability to retain sharks and reduce gear loss (Gilman et al. 2008). Trips targeting sharks were longer (average 14.4 days ± 7.5; 1−49) than those for dolphinfish, (average 7.5 days ± 2.2; 2−15). Both fisheries used Humboldt squid (*Dosidicus gigas*), flying fish (*Exocoetus volitans*), and chub mackerel (*Scomber japonicus*) as bait. Porcupinefish (*Diodon hystrix*), Peruvian Pacific sardine (*Sardinops sagax sagax*), and small cetacean meat (mostly bottlenose dolphin (*Tursiops truncatus)* and long-beaked common dolphin (*Delphinus capensis*); an illegal practice in Peru) were also used as bait during the shark season (Mangel et al. 2010).

Catch per unit effort (CPUE) is reported as number of sharks caught per 1000 hooks, and data are represented as means ± standard deviation (range). All spatial analyses and maps were created using ESRI ArcMap 10. A fishnet grid of 2500 km^2^ cells was used to generate spatially explicit data. Fork length (FL) was calculated from total length (TL) for use in comparing the fork lengths measured in observed catch to the legal minimum landing size using the equations: FL = 0.821 + 0.911(TL) for mako sharks and FL = −1.615 + 0.838(TL) for blue sharks (Francis and Duffy 2005). All statistical tests were carried out using the software R v.3.0.2 (R Development Core Team 2010). A one-sample proportions test with continuity correction was carried out to calculate confidence intervals for sex ratios observed within species. For temporal analysis of fork length, we used general linear models (GLMs) with log transformation, where fork length was the dependent variable with sex and season as factors. Shark catch data were zero-inflated, therefore making a poisson error structure invalid, resulting in the use of a negative binomial GLM, which include fixed effects (Year and Season) as well as an offset term for fishing effort, where Hooks was representative of an increase in sharks caught by increments of 1000 hooks of effort. This use of the log offset allows the intercept parameters estimated by the GLM to be interpreted as catch per unit effort. The dependent variable was the total count of sharks captured during a given fishing set. Using the GLM, we were able to calculate the catch for every 1000 hooks deployed. This was accomplished using the means from the model output to derive the catch per unit effort. The negative binomial GLM was fitted using the MASS package for R v. 3.0.2 (R Development Core Team 2010).

## Results

### Spatial patterns

**Figure 1:**
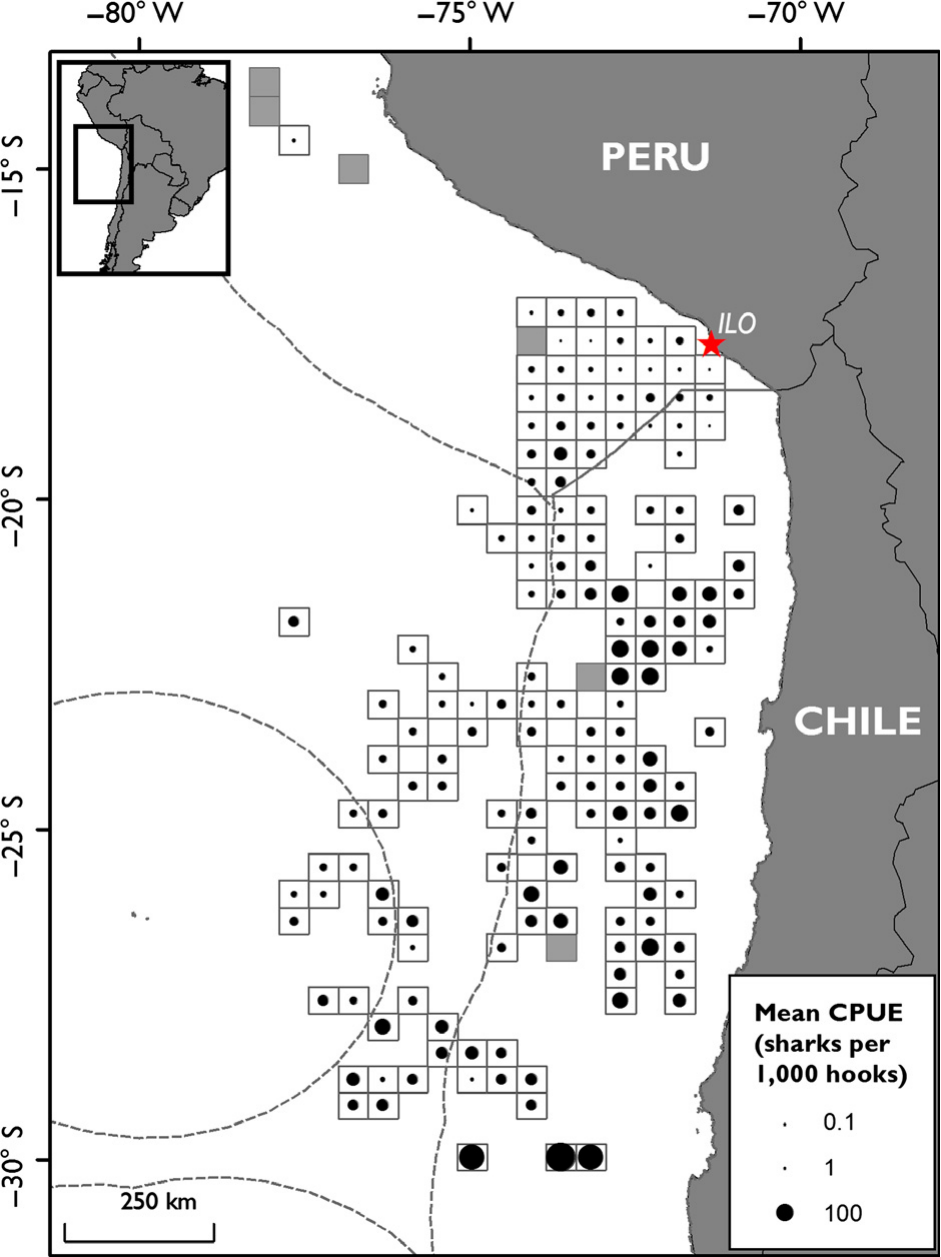
Average catch per unit effort (CPUE; sharks per 1000 hooks) within grid cells of 2500 km^2^ represented by black dots. Gray-shaded grid cells represent areas that were fished, but yielded zero catch. Dashed gray lines represent the EEZs of Peru and Chile, recently agreed between the two countries (Claus et al. 2014, *Flanders Marine Institute*; *VLIZ*).

A diffuse pattern of CPUE emerges for this fishery (Fig. 1), showing little concentration in specific fishing areas, with trips of high catch rates of sharks spread over the entire fishing area. There is a high proportion of effort along the Peruvian-Chilean Economic Exclusive Zone (EEZ) border, with low catch rate. Higher success is found in higher-latitude Chilean waters, with few fishing trips resulting in zero catch (Fig. 1).

### Species composition

A total of 16,610 sharks were landed by the observed vessels with eight shark species identified (Table S1). Blue sharks accounted for 70.6% of all sharks caught, mako sharks 28.4%, and other species 1% (Table S1, S4). Ray species, comprising mostly of *Dasyatis* spp., are also caught within these fisheries, but are discarded. The heads of sharks and viscera are also discarded due to storage space constraints, with the rest of the shark retained.

### CPUE

**Figure 2:**
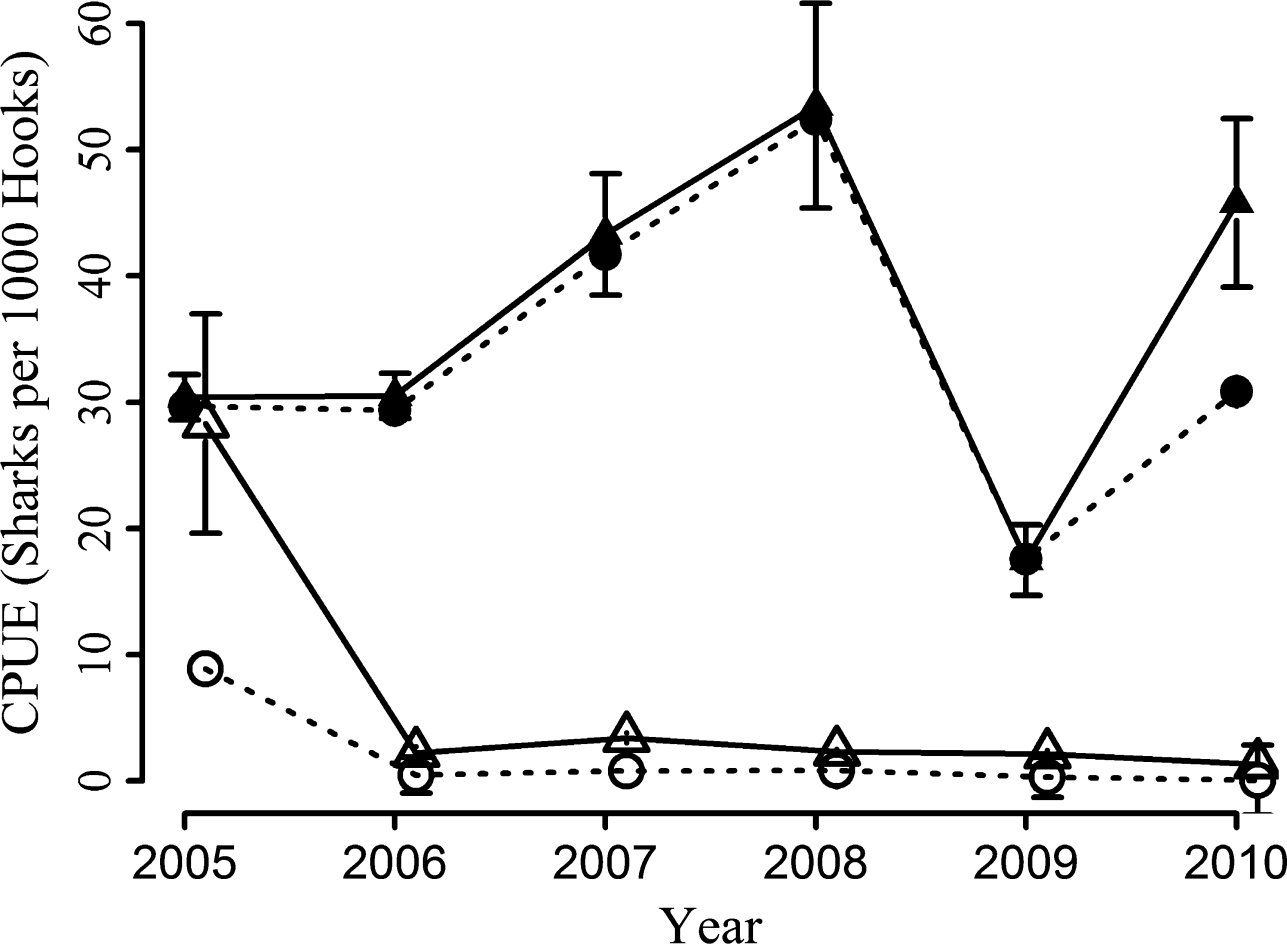
Average CPUE (sharks per 1000 hooks) per year. Nominal CPUE values are plotted for shark season (filled circles) and dolphinfish season (open circles) with unbroken lines. Standardized CPUE values from GLM analysis are plotted for shark season (filled triangles) and dolphinfish season (open triangles) with dashed lines. Standard error bars are shown.

Shark catch is spread throughout the year, with sharks being caught in every month (Fig. S2). The longer shark season contributes the majority of the effort observed, with 57.9% of the total number of trips and 60.1% of the sets observed. Within this fishery, 84 trips were observed with 618 sets, deploying 462,438 hooks (Table S2, Fig. S3) with 579 sets (93.7%) resulting in shark capture. A CPUE of 33.6 ± 10.9 sharks per 1000 hooks was calculated (Fig 2, Table S3). CPUE appears to remain at a relatively constant rate, month to month, with sporadic sets that return higher rates of shark catch (Fig. S2). Shark catch in the dolphinfish season is incidental, but all sharks are retained. During observation of 61 trips, 402 sets and 283,446 hooks were deployed (Table S2), with 98 sets resulting in shark catch (24.4%), resulting in a CPUE of 1.9 ± 3.1 sharks per 1000 hooks (Fig. 2, Table S3). There were occasional sets that returned very high catches of sharks, with three sets in 2005 catching over 150 sharks per set (Fig. S2a).

### Size composition

**Figure 3:**
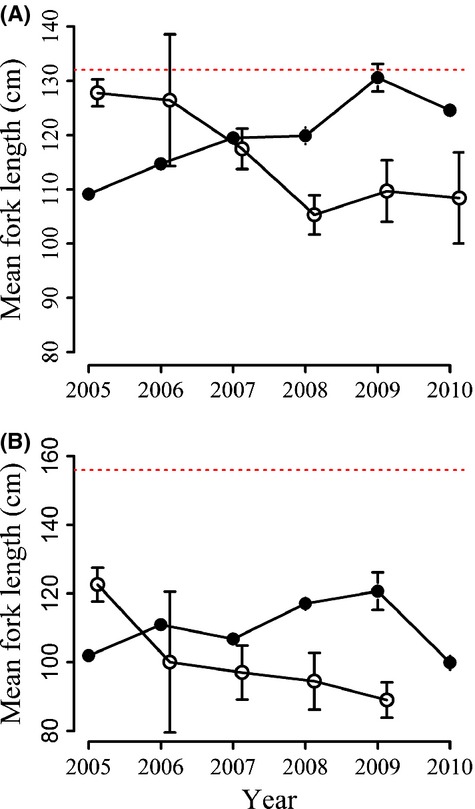
Mean fork length for blue (A) and mako (B) sharks divided by shark season (filled circles) and dolphinfish season (open circles). Dashed line represents the legal minimum landing size for the species. Standard error bars are shown.

Average fork length was 115.8 cm ± 8.7 (105.3−127.3) for blue sharks and 99.5 cm ± 10.9 (89−122.6) for mako sharks within the dolphinfish season. During the shark season, average fork length was 119.9 cm ± 5.2 (109.7−130.6) for blue sharks and 109.5 cm ± 7.4 (100−120.7) for mako sharks (Fig. 3, Table S6). There was a significant difference in mean fork lengths for blue sharks between sexes (GLM; *F*_1,4113_ = 4.71, *P* < 0.05), with larger mean fork length in males during the shark season, but no significant difference between sexes or season in fork length for mako sharks (GLM; *F*_1,1736_ = 0.04, *P* > 0.1). For the shark season, an average of 40% ± 12 (27−61) of all sharks captured during the study were measured. An average of 74.7% ± 7.3 (64.5−85.2) of all sharks were under the MLS (blue sharks: 71.1% ± 6.8 (60.6−80); mako sharks: 88.6% ± 13 (63.4−100); Fig. S1, Table S5). For the dolphinfish season, an average of 82% ± 35% (5−100) of all sharks captured were measured and an average of 85% ± 9.7 (73−100) were below MLS (blue sharks: 78.6% ± 12.9 (62.5−100); mako sharks: 98.9% ± 2.2 (94.4−100); Fig S1, Table S5). There was some seasonal variation, however, for one species. The majority of blue sharks caught during the first quarter of the year (January–March) were above the legal MLS for both sexes, with this proportion decreasing throughout the year (Fig. S4). There was no significant deviance from a 1:1 male to female ratio for either species in either fishery (one-sampled proportion test; blue sharks; χ^2^ = 0.02, *P* > 0.5, mako sharks; χ^2^ = 1.06, *P* > 0.1).

## Discussion

Blue and mako sharks have a pan-Pacific distribution, with tagging studies providing evidence of wide movement throughout the Pacific (Sippel et al. 2011; Abascal et al. 2011); however, no tagging data have yet demonstrated movement across the equator (Weng et al. 2005; Stevens et al. 2010; Sippel et al. 2011). Consensus within the International Scientific Committee (ISC) of the Shark Working Group supports two sub-populations for each of these species in the North Pacific, distinct from the South Pacific demarked by the equator, although more information is needed to further explore the potential for size and sex segregation as proposed by Nakano (1994). In this study, we have taken major steps forward in beginning to understand some of the patterns of exploitation of the southern stocks.

This study provides the most in-depth analysis of the Peruvian shark fishery to date and underlines the power of using onboard observers in the fisheries sector to obtain detailed information. Given the importance of SSF in Peru (Alfaro-Shigueto et al. 2010; Estrella Arellano and Swartzman 2010), this work highlights areas of possible concern. The number of Peruvian longline vessels involved in the SSF fleet was shown to have increased by >350% between 1995 and 2005, conducting an estimated 11,316 trips in 2002 (in Alfaro-Shigueto et al. 2010), representing a 54% increase over the preceding decade (Estrella Arellano and Swartzman 2010). Alfaro-Shigueto et al. (2010) estimated up to 80 million hooks are set per annum by Peruvian longline vessels. To place these data in a global context, this number of hooks is equal to a third of the global swordfish longline fishery and double the total Hawaiian longline fleet (Lewison et al. 2004). Using global landings of shark weight to calculate national contribution to global shark landings, Lack and Sant (2011) concluded that the entire Peruvian fishery is responsible for 1.2% of global shark catch. Based on recent global estimates of shark take reaching 100 million (range: 63–73 million) sharks caught annually (Worm et al. 2013), the Peruvian SSF would represent some 1.2 million sharks being caught. If the observed catch rates in this study period are representative of the national longline fleet, it is likely that the longline SSF alone is catching in excess of this estimate. Overall national catch figures would be greatly increased when considering the effort of an extensive, yet poorly studied, small-scale gillnet fishery that also operates in Peru (setting over 100,000 km of nets per annum) specifically targeting sharks and rays (Alfaro-Shigueto et al. 2010).

The CPUE observed in this study (33.6 sharks per 1000 hooks) is higher than many other reported fisheries in the South Pacific region highlighting the biodiversity importance of the region, despite heavy fishing effort. The Chilean dolphinfish and shark fishery caught 24 sharks per 1000 hooks, with the Mexican Pacific (Velez-Marin and Marquez-Farias 2009; Smith et al. 2009), and Papua New Guinean (Kumoru 2003) fisheries catching less than one shark per 1000 hooks (Bizarro et al. 2009). The Costa Rican longline fishery catches high numbers of silky sharks (*Carcharhinus falciformis*) as bycatch, with rates of 2.96 sharks per 1000 hooks (Whoriskey et al. 2011) and 8.08 sharks per 1000 hooks (Dapp et al. 2013) reported. A review of the Brazilian commercial longline fishery (southwest Atlantic) showed higher, but comparable CPUE values to our study, reporting 38.3 blue sharks per 1000 hooks (Montealegre-Quijano and Voreen 2010). This study also demonstrated a dominance of blue sharks and was conducted within similar latitudes, suggesting the possibility of a conspecific niche occupied by blue sharks in the Atlantic Ocean.

Looking into the impact of the Peruvian fishery, removal of large, oceanic predators has been shown to cause deleterious effects on the ecosystems they inhabit, with the potential to cause trophic cascades (Stevens et al. 2000; Myers et al. 2007; Heithaus et al. 2008; Baum and Worm 2009). Given the rapid expansion and growth of Peru's longline fisheries along with high CPUE rates of largely juvenile sharks, it may be that fisheries sustainability comes into question. What has the impact been thus far? Prolonged exposure to fishing pressure has been shown to alter size and age structure within populations (Law 2000; Jackson et al. 2001) with historical data showing that almost all fisheries start out harvesting larger individuals (Jennings and Kaiser 1998). There are recent studies elsewhere that show juvenile and sexually immature individuals are being caught in high numbers (USA: Ward and Myers 2005; Mexico: Bizarro et al. 2009; Cartamil et al. 2011; Costa Rica: Dapp et al. 2013; Chile; Bustamante and Bennett 2013). Powers et al. (2013) examined the changes in size and species winning in fishing rodeos in the Gulf of Mexico between 1929 and 2009. Size of sharks caught was shown to increase until the 1980s and then showed a 50–70% decline with a shift toward smaller shark species, with none of the tiger sharks (G*aleocerdo cuvier*) caught in the last 20 years considered sexually mature. They suggest the increase in longline fishing activity from the 1990s as the cause of such a decline in size. Ward and Myers (2005) showed a decrease in weight of caught blue (52–22 kg, approx. 2 m to 1.52 m fork length) and mako sharks (74–38 kg, approx. 1.88 m to 1.52 m fork length) between the 1950s and the 1990s. Dapp et al. (2013) showed that the Costa Rican fishery, mainly consisting of silky sharks, experienced higher catch rates further from shore, with catches comprising heavily of juvenile or sexually immature individuals. These authors hypothesized that it is likely that continued fishing pressure is to blame for the removal of large individuals from the population.

It seems likely, although we cannot be certain, that there has been a fishing pressure induced reduction in size in our study populations, but quantifying the changes requires prior information (Jackson et al. 2001), which we do not have for our study populations. We are, however, now in possession of excellent baselines for future reference in this region. Additionally, fisheries can induce changes in fish life history (Hutchings et al. 2012). Hoenig and Gruber (1990) suggested that exploitation of a population could result in increased growth rates, increased fecundity, but reductions in mean age, mean size, proportion of gravid females, and a reduction in age at maturity in response to increased fishing pressure. This would explain maintenance of catch rates with an increasing predominance of smaller individuals (Law 2000). A final factor that must be considered, however, for high prevalence of immature individuals within catches observed is that the surrounding area serves as a nursery ground (Springer 1967; Cartamil et al. 2011). To gain insights into these issues, more detailed work would be needed on reproductive status, to determine clasper length and development and presence of gravid females.

Our current study shows that although a mandated size limit is in place, these fishermen appear unable to catch individuals of this size, suggesting that no size-selective fishing is taking place. Gear type has remained constant, and therefore, selectivity for size class has not changed, supporting the premise that the largest individuals have been fished out, shifting population structure to a smaller body size or shifting the fishing pressure to a smaller size of individuals. Exploitation patterns associated with high-proportional fishing mortality of immature fish can have a significant negative effect on current stock status, providing empirical support for the “spawn-at-least-once” principle (Vasilakopoulos et al. 2011). This fishery would therefore appear to require mitigation strategies be put in place to try and allow for breeding to occur.

From a policy perspective, this indicates the limited impact protective legislation has had in the absence of adequate enforcement. Similar constraints have been shown in Peru for the bycatch of cetaceans (Mangel et al. 2010) and turtles (Alfaro-Shigueto et al. 2011). Given these fisheries are large, widespread, increasing in magnitude, using highly effective gear and retaining all sharks regardless of fishing season, there is a clear need for monitoring and multi-national cooperation. Fishing effort spans multiple geo-political zones and implementation of appropriate legislation for the maintenance of food security, fisher livelihoods and the management and conservation of vulnerable species such as sharks is critical. We need to assess the catch composition at a greater number of representative ports along the Peruvian coast toward improving our understanding of spatial, inter- and intra-species catch rates, and regional and national patterns of catch and species distribution. Investigations of size distributions and changes in size over longer periods of time are needed. In order to fully understand the effectiveness of any mitigation, further biological information is needed, with reproductive state of individuals caught being a constraint of this study as the majority of catch were immature.

Strategies such as restrictions on the number of fishing permits, number of hooks set, specific fishing grounds, and total allowable catch limits have shown some success in countries such as Papua New Guinea (Kumoru 2003) and Mexico (Cartamil et al. 2011). Challenges with enforcement and implementation of similar measures in Peru will likely continue, given the remote location of these fishing grounds. Implementation and enforcement of legislation within SSF have proven to be challenging (Salas et al. 2007). We suggest that the completion of Peru's national plan of action (NPOA) for conservation and management of sharks is of the highest importance to create multinational links and establish guidelines on best practices to conserve sharks and promote sustainable fisheries. This work is extremely timely as recent media coverage of fishing activities in this region has shed light on the current state of these fisheries and the government of Peru has deemed it necessary to complete a national plan of action. Findings from the current study will aid in the assessment of current management strategies in place for sharks, and amendments and additional regulations should be developed with the aim of conserving these shark populations.

## Supporting Information

**Table S1.** Shark species identified from observed trips (*N* = 145) during the study period of 2005–2010 within dolphinfish and shark fishing seasons embarking from the port of Ilo, Peru (IUCN 2001). **Table S2.** Effort data for the study period. **Table S3.** Catch data for the study period. **Table S4.** Species identification data for the study period. **Table S5.** Species, size and sex composition data for the study period. **Table S6.** Mean fork lengths (cm) for each fishing season (dolphinfish; shark), species (blue; mako) and sex (male; female) for each year of observer data. **Table S7.** Hook dimensions (mm) used within dolphinfish and shark seasons. **Figure S1.** Stacked bar chart showing fork length frequencies for shark season (gray) and dolphinfish season (white) split by species (mako sharks; A & B; blue sharks; C & D) and sex (males; A & C, females; B & D). Dashed lines denote legal minimum landing size for each species. (N)umber of sharks identified to sex and species level and measured are shown. **Figure S2.** CPUE per month for 2005–2010 (A–E), split by shark and dolphinfish seasons. **Figure S3.** Effort, split into total number of trips (top), total number of sets (middle) and total number of hooks (bottom) across each year, split by shark (filled circles) and dolphinfish (open circles) seasons. **Figure S4.** Fork length frequencies for each quarter of the year split by species and sex.
