# Supplementary material for: Big catch, little sharks: Insight into Peruvian small-scale longline fisheries
Source: Ecol Evol. 2014 May 14;4(12):2375–83. doi: 10.1002/ece3.1104 (PMC4203286; doi:10.1002/ece3.1104)
Supplement: Supplementary file 1 [file ece30004-2375-sd1.docx]

**Supplementary material**

**Table S1.** Shark species identified from observed trips (N=145) during the study period of 2005-2010 within dolphinfish and shark fishing seasons embarking from the port of Ilo, Peru (IUCN 2001). IUCN classification status’ (DD=Data Deficient; LC=Least Concern; VU=Vulnerable; NT=Near Threatened; EN=Endangered) are shown.

| **Species Name** | **Scientific Name** | **IUCN Status** | **Dolphinfish season** | **Shark season** |
| --- | --- | --- | --- | --- |
| Blue | *Prionaceglauca* | NT | X | X |
| Shortfinmako | *Isurusoxyrinchus* | VU | X | X |
| Porbeagle | *Lamnanasus* | VU |  | X |
| Common thresher | *Alopiasvulpinus* | VU |  | X |
| Smooth hammerhead | *Sphyrnazygaena* | VU | X | X |
| Carcharhinusspp (bronze whaler, silky, tope) | *Carcharhinusbrachyurus, Carcharhinusfalciformis*  *Galeorhinusgaleus* | NT,  NT (VU^*^)  VU |  | X |
| Smoothhoundspp | *Mustelusspp* | VU |  | X |

^*^Silky shark classified as vulnerable in the eastern Pacific.

**Table S2.**Effort data for the study period.Annual means by fishing season (dolphinfish; shark); mean total number of sets and trips, with averages of hooks per set and trip. All data displayed as mean ± S.D (range; N).

|  | **Dolphinfish season** | **Shark season** |
| --- | --- | --- |
| **Total Trips** | 61 | 84 |
| **Total Sets** | 402 | 618 |
| **Mean Sets per trip** | 7.4 ± 1.6 (31-139; 402) | 7.1 ± 0.9 (17-215; 618) |
| **Mean Hooks per set** | 676.6 ± 64.6 (400-1500; 2.8x10⁵) | 705.1 ± 89.8 (260-1700; 4.6x10⁵) |
| **Mean Hooks per trip** | 4903 ±785 (650-9440; 2.8x10⁵) | 4983±899 (800-11550; 4.6x10⁵) |

**Table S3.** Catch data for the study period. Annual means by fishing season (dolphinfish; shark), mean total number of individual sharks caught, average number of individual sharks caught per trip and set, and nominal and standardised CPUE as number of sharks per 1,000 hooks. All data displayed as mean ± S.D (range; N).

|  | **Dolphinfish season** | **Shark season** |
| --- | --- | --- |
| **Mean Sharks Caught per Year** | 181.8 ± 361  (0-988; 1091) | 2586.5 ± 1739.7  (202-5269;15519) |
| **Mean No. Sharks per Set** | 1.4 ± 2.6 (0.205; 402) | 24 ± 8.7 (0-324; 618) |
| **Mean No. Sharks per Trip** | 8.4 ± 13.3 (0-494; 61) | 169.2 ± 59.1 (0-988; 84) |
| **Nominal Mean CPUE (sharks per 1,000 hooks)** | 3 ± 20.7 (0-256.3; 402) | 33.2 ± 35.6 (0-294.6; 618) |
| **Standardised CPUE (sharks per 1,000 hooks)** | 1.9 ± 3.1 (0.05-8.9; 402) | 33.6 ± 10.9 (17.6-52.4; 618) |
| **No. Positive Sets** | 98 | 579 |
| **Prop Positive Sets From Total** | 24.4 | 93.7 |

**Table S4.**Species identification data for the study period. Means by fishing season (dolphinfish; shark); mean number of individual sharks identified to species level from total catch, with proportions of blue, mako and other sharks identified to species level within total catch for the sampled sets. All data displayed as mean ± S.D (range; N)

|  | **Dolphinfish season** | **Shark season** |
| --- | --- | --- |
| **Mean No. Species ID** | 25.3 ± 21.3 (0-108; 151) | 1793.5 ±1177.2 (0-4106; 5889) |
| **Prop Total Captured** | 0.8 ± 0.4 (0.1-1; 1091) | 0.7 ± 0.2 (0.4-1; 1.6x10⁴) |
| **Prop Blue** | 0.7 ± 0.3(0.03-1; 108) | 0.3 ± 0.1(0.2-0.5; 4106) |
| **Prop Mako** | 0.2 ± 0.1 (0-0.3; 40) | 0.1 ± 0.04(0.1-0.2; 1741) |
| **Prop Other** | 0.01 ± 0.03(0-0.17; 3) | 0.002±0.002(0.0-0.004; 42) |

**Table S5.**Species, size and sex composition data for the study period. Means by season (dolphinfish; shark), proportions of blue and mako sharks measured and sexed from sampled sets, proportions of blue and makofemales, mean fork lengths for blue and mako sharks and proportions of measured blue and mako sharks under the legal minimum landing size. All data displayed as mean ± S.D (range; N).

|  | **Dolphinfish season** | **Shark season** |
| --- | --- | --- |
| **Prop Female Blue** | 0.7 ±0.28(0.2-1; 104) | 0.5 ± 0.04(0.4-0.6; 4018) |
| **Prop Female Mako** | 0.7 ± 0.4(0-1; 42) | 0.5 ± 0.1(0.5-0.6; 1710) |
| **Prop < MLS Blue** | 0.8 ± 0.1 (0.6-1; 107) | 0.7 ± 0.1 (0.6-0.8; 4095) |
| **Prop < MLS Mako** | 1.00 ± 0.02(0-1; 43) | 0.9 ± 0.1(0.6-1; 1705) |
| **Mean Length Blue** | 115.8±8.767-177; 107) | 119.9±5.2(109.7-130.6; 4095) |
| **Mean Length Mako** | 99.5±10.1(52-163; 43) | 109.5±7.4(100-120.7; 1705) |

**Table S6.** Mean fork lengths (cm) for each fishing season (dolphinfish; shark), species (blue; mako) and sex (male; female) for each year of observer data.

|  | Season | Species | Sex | 2005 | 2006 | 2007 | 2008 | 2009 | 2010 |
| --- | --- | --- | --- | --- | --- | --- | --- | --- | --- |
|  | Dolphinfish | Blue | Male | 128.8 | 121.9 | 123.8 | 102.8 | NA | 120.3 |
|  |  |  | Female | 124 | 135.5 | 116.4 | 107.8 | 109.7 | 96.5 |
|  |  | Mako | Male | 125.4 | NA | 98.8 | 74.7 | NA | NA |
|  |  |  | Female | 118.9 | 93.8 | 104.5 | 89 | NA | NA |
|  | Shark | Blue | Male | 110.1 | 114.7 | 119.8 | 127 | 134.9 | 128.4 |
|  |  |  | Female | 111.6 | 115.4 | 119.1 | 113.7 | 125.6 | 121.5 |
|  |  | Mako | Male | 103.2 | 113.7 | 105 | 119 | 120.8 | 99.1 |
|  |  |  | Female | 101 | 110 | 108.6 | 115.3 | 120.6 | 100.3 |

**Table S7.** Hook dimensions (mm) used within dolphinfish and shark seasons. Total length (TL), measured from the eye of the hook to the lowest point on the bend of the hook. Gape, measured as the narrowest part of the hook from the point to the shank.

| **Hook type** | **Total length**  **(TL; mm)** | **Gape**  **(mm)** | **Shark season** | **Dolphinfish season** |
| --- | --- | --- | --- | --- |
| **J1** | 91.02 | 29.95 | X |  |
| **J2** | 81.93 | 25.74 | X |  |
| **J3** | 73.26 | 23.55 |  | X |
| **J4** | 65.12 | 19.95 |  | X |
| **J5** | 57.68 | 19.56 |  | X |


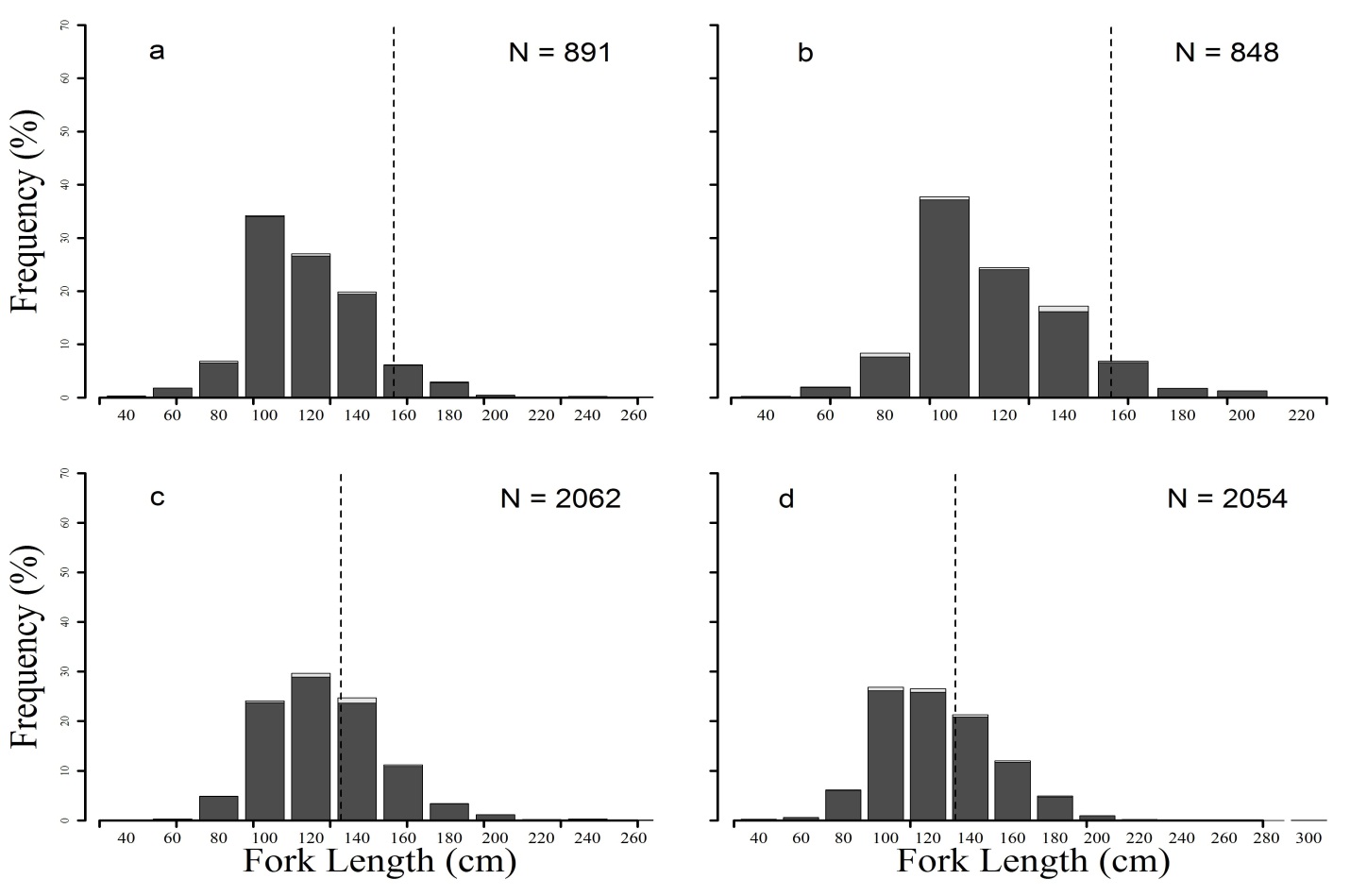


^Figure S1. Stacked bar chart showing fork length frequencies for shark season (grey) and dolphinfish season (white) split by species (mako sharks; a & b; blue sharks; c & d) and sex (males; a & c, females; b & d). Dashed lines denote legal minimum landing size for each species. (N)umber of sharks identified to sex and species level and measured are shown.^

^
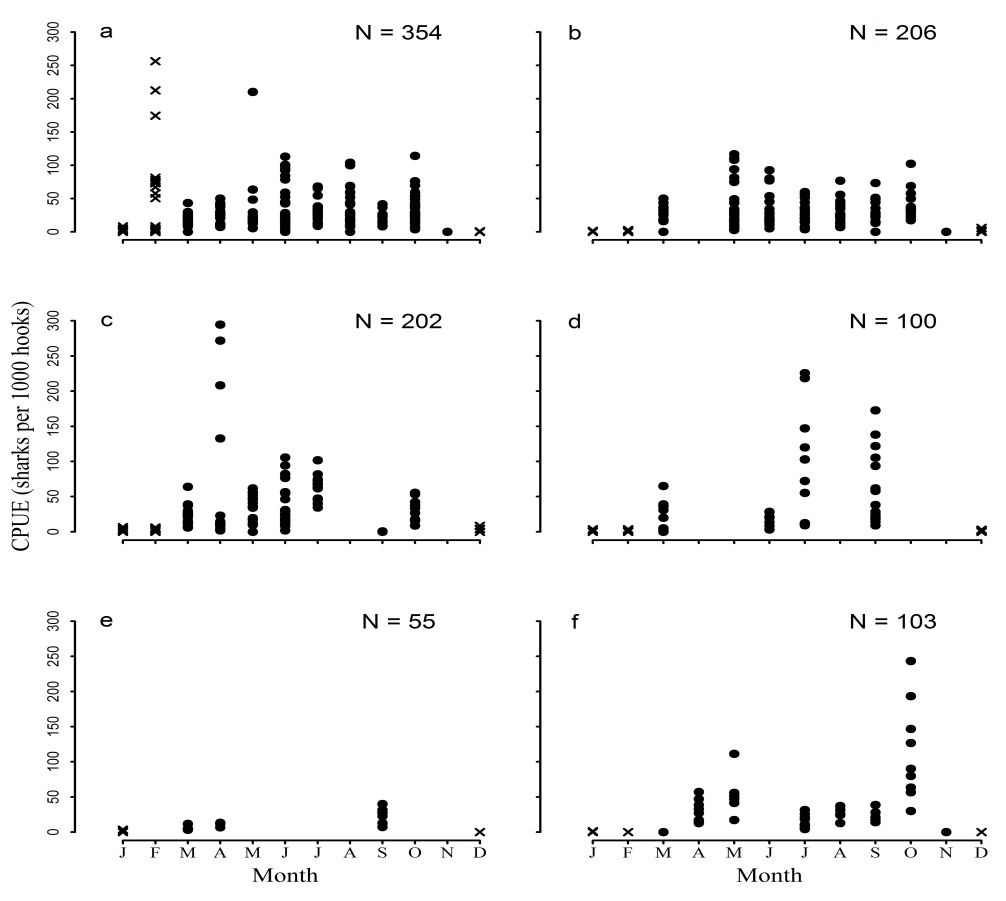
^

_Figure S2. CPUE per month for 2005 – 2010 (a – e), split by shark and dolphinfish seasons. Each filled circle (shark season) and cross (dolphinfish season) represents an individual set within each month and its associated catch rate. (N)umber of sets is shown._


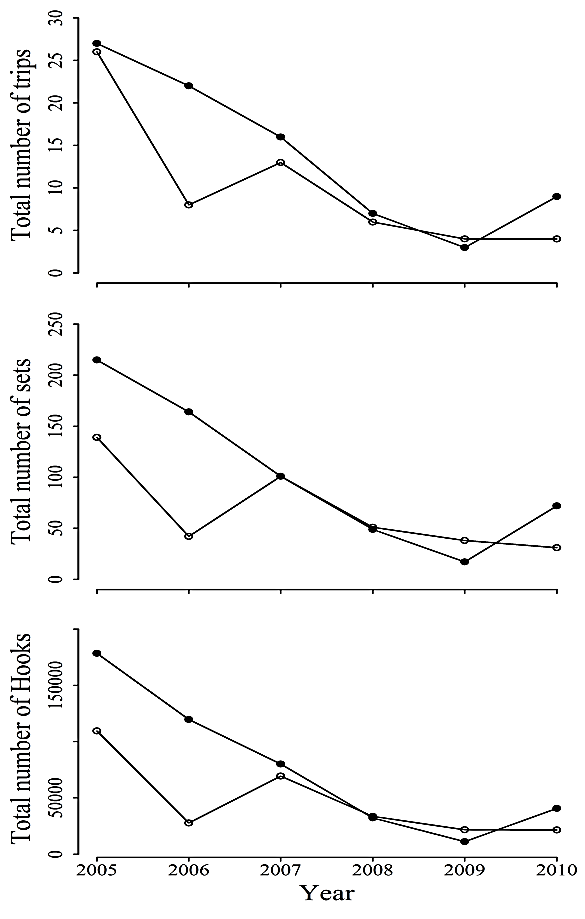


_Figure S3. Effort, split into total number of trips (top), total number of sets (middle) and total number of hooks (bottom) across each year, split by shark (filled circles) and dolphinfish (open circles) seasons._

_
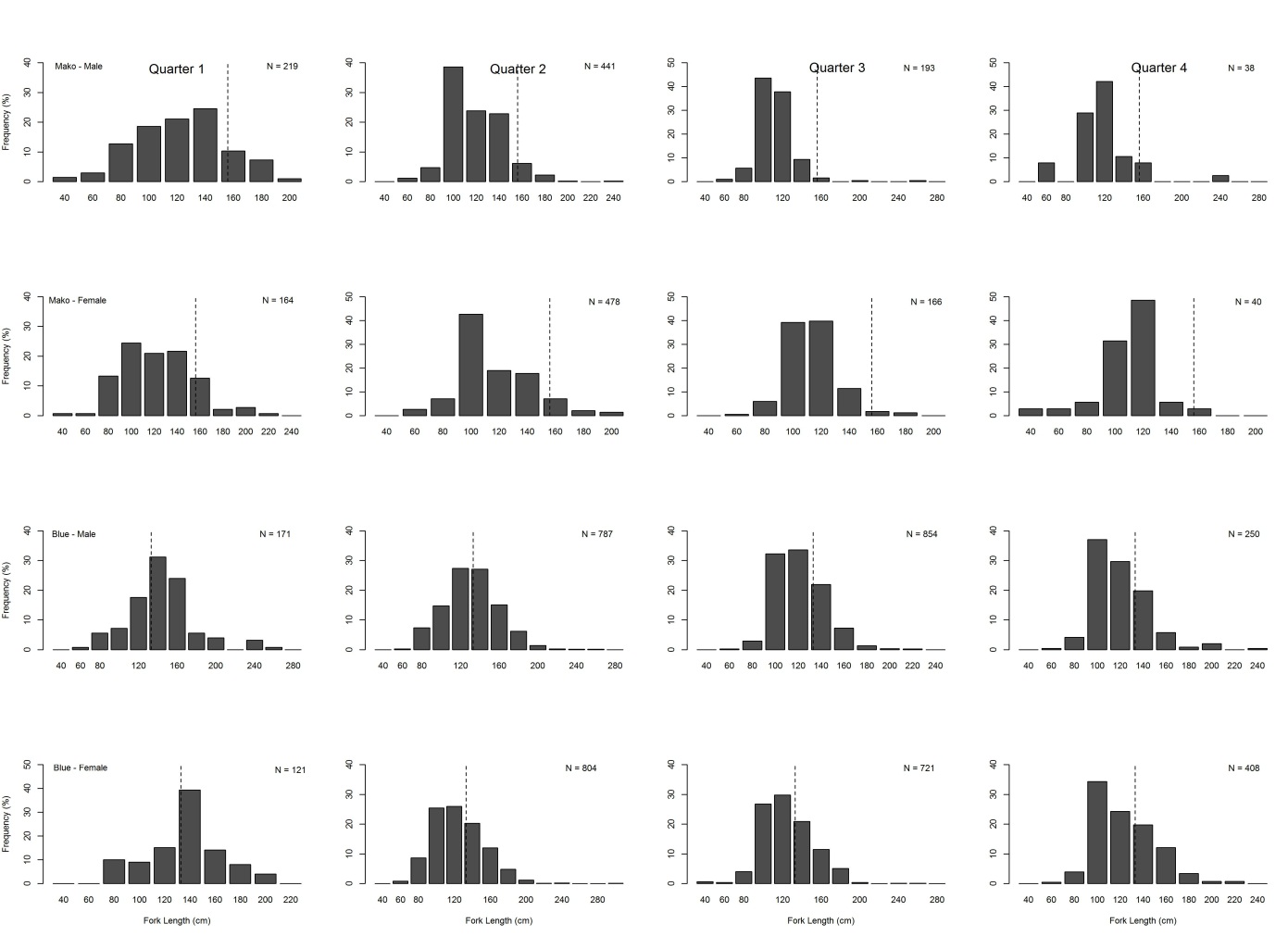
_

_Figure S4. Fork length frequencies for each quarter of the yearsplit by species and sex. Dashed line represents the legal minimum landing size for each species. (N)umber of sharks identified to sex and species level and measured are shown._

_References:_

IUCN (2001). IUCN red list categories and criteria: version 3.1. IUCN Species Survival Commission, Gland Switzerland and Cambridge, U.K. p. 38**.**
